# Misplacement of left ventricular vent into the aortic root during a re-do Bentall procedure: a case report

**DOI:** 10.1186/s40981-023-00608-w

**Published:** 2023-03-21

**Authors:** Taisuke Kumamoto, Chieko Hiraoka, Kotaro Murakami, Miki Fujita, Yuji Kunitoku, Kiyohiko Kato

**Affiliations:** Department of Anesthesiology, Saiseikai Kumamoto Hospital, 5-3-1, Chikami, Minami-ku, Kumamoto, 861-4193 Japan

**Keywords:** Left ventricular vent, Misplacement, Transesophageal echocardiography, Aortic root, Cardiopulmonary bypass

## Abstract

**Background:**

The left ventricular (LV) vent is commonly inserted via the right superior pulmonary vein (RSPV) and directed toward the LV cavity through the mitral valve. We report a rare case in which the tip of the LV vent was misplaced into the aortic root across the aortic valve.

**Case presentation:**

An 88-year-old man was scheduled to undergo the Bentall procedure. After initiation of cardiopulmonary bypass, the LV vent was inserted via the RSPV. Anterograde cardioplegia was administered via the aortic root cannula after the ascending aorta was cross-clamped. The electrocardiogram did not result in complete cardiac arrest, even after delivery of two-thirds of the planned dose. A transesophageal echocardiographic examination showed that the tip of the LV vent was misplaced into the aortic root across the aortic valve.

**Conclusions:**

It is important to confirm the tip position by transesophageal echocardiography to prevent severe complications associated with the LV vent.

**Supplementary Information:**

The online version contains supplementary material available at 10.1186/s40981-023-00608-w.

## Background

Left ventricular (LV) venting is performed during cardiac surgery to remove air from the left heart chambers, ensure an adequate surgical field of view, and protect the myocardium by preventing LV dilatation [1]. The LV vent is commonly inserted via the right superior pulmonary vein (RSPV) and directed toward the LV cavity through the mitral valve [2].

Misplacement of the LV vent may cause insufficient venting [2]. Endocardial compression by the LV vent may cause LV lacerations, thus leading to formation of an LV pseudoaneurysm [3]. It is recommended to confirm the final position of the LV vent by transesophageal echocardiography (TEE) [3]; however, it may be difficult to visualize the position of the tip in the mid-esophageal four-chamber view.

Herein, we report a rare case in which the tip of the LV vent inserted via the RSPV was misplaced into the aortic root across the aortic valve despite confirmation of the LV vent by TEE.

## Case presentation

We have obtained written informed consent from the patient for publication of this case report.

**Fig. 1:**
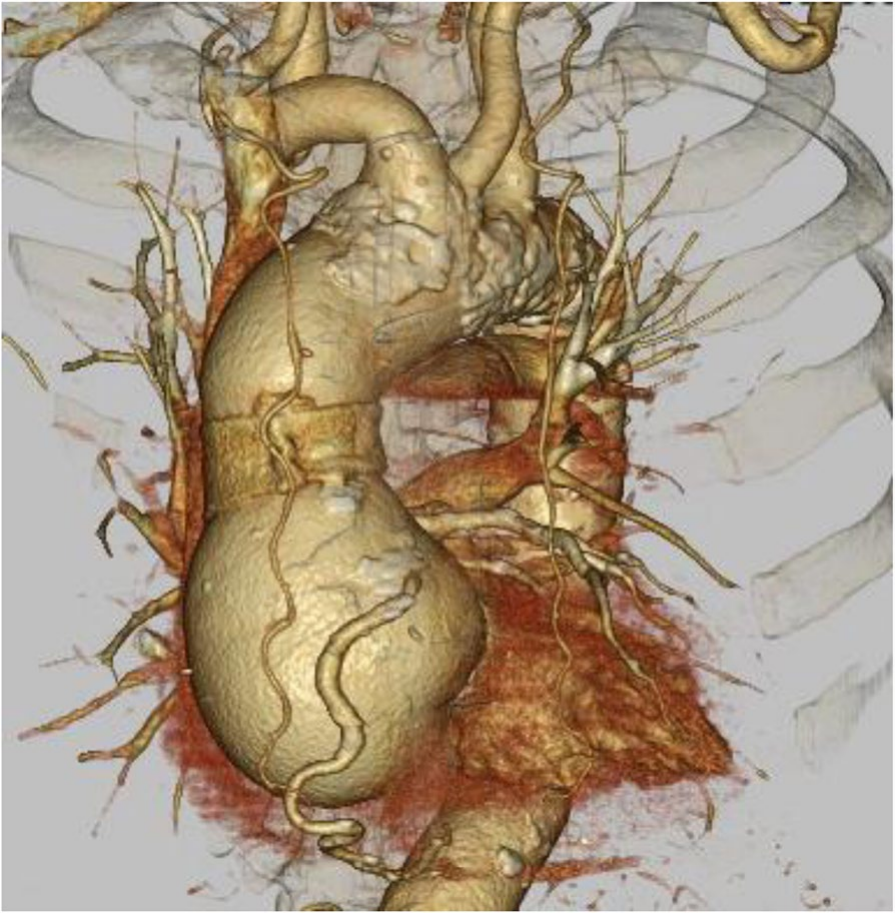
Preoperative three-dimensional computed tomography. The sinus of Valsalva was 62 mm in diameter

The patient was an 88-year-old man with a height of 156 cm and a body weight of 48 kg. His medical history included hypertension and hyperlipidemia. He had undergone aortic valve replacement with a biological valve (Crown PRT^®^; Sorin Group, Burnaby, Canada) for aortic regurgitation (AR) associated with annulo-aortic ectasia 4 years previously. The sinus of Valsalva diameter had increased from 55 to 62 mm in 6 months based on computed tomography during follow-up for annulo-aortic ectasia (Fig. 1); therefore, he was scheduled to undergo the Bentall procedure via a repeat sternotomy.

At the time of admission, a transthoracic echocardiography revealed mild AR with an LV ejection fraction of 63% and LV end-diastolic diameter of 41 mm. Coronary angiography did not demonstrate significant stenosis, and an electrocardiogram showed a normal sinus rhythm. Laboratory blood test results were within normal ranges, except for a creatinine level of 1.21 mg/dL.

On arrival in the operating room, standard monitors were placed, and the left radial artery was cannulated for arterial blood pressure monitoring. General anesthesia was induced with 2 mg of midazolam, 500 μg of fentanyl, and 50 mg of rocuronium, and anesthesia was maintained with propofol, fentanyl, and rocuronium. After tracheal intubation, a central venous catheter was placed via the right internal jugular vein. TEE showed sinus of Valsalva enlargement and mild AR.

Following a standard re-sternotomy, the adhesions between the sternum and the aortic artery were exfoliated to expose the surgical field. In preparation for cardiopulmonary bypass (CPB), arterial and venous cannulas were inserted in the right femoral artery and vein, respectively. After initiation of CPB, the LV vent (Edwards Lifesciences, Irvine, CA, USA) was inserted via the RSPV. The LV vent was inserted by surgical palpation on the first attempt without difficulty. Insertion of the vent was confirmed by observing the presence of ventricular arrhythmias and regurgitation of high-pressure pulsatile blood flow from the LV vent. The LV vent was shown on the mid-esophageal four-chamber view to be inserted into the LV cavity through the mitral valve; however, the tip of the LV vent was not visualized.

After systemic cooling was initiated, the ascending aorta was cross-clamped, and pure crystalloid cardioplegic solution (Miotecter^®^; Mochida Pharmaceutical Co., Ltd., Tokyo, Japan) was administered in an antegrade direction through the aortic root cannula, which had been placed proximal to the cross-clamped aorta. The plan was to administer 1500 mL of initial cardioplegia at a rate of 250–400 mL/min.

**Fig. 2:**
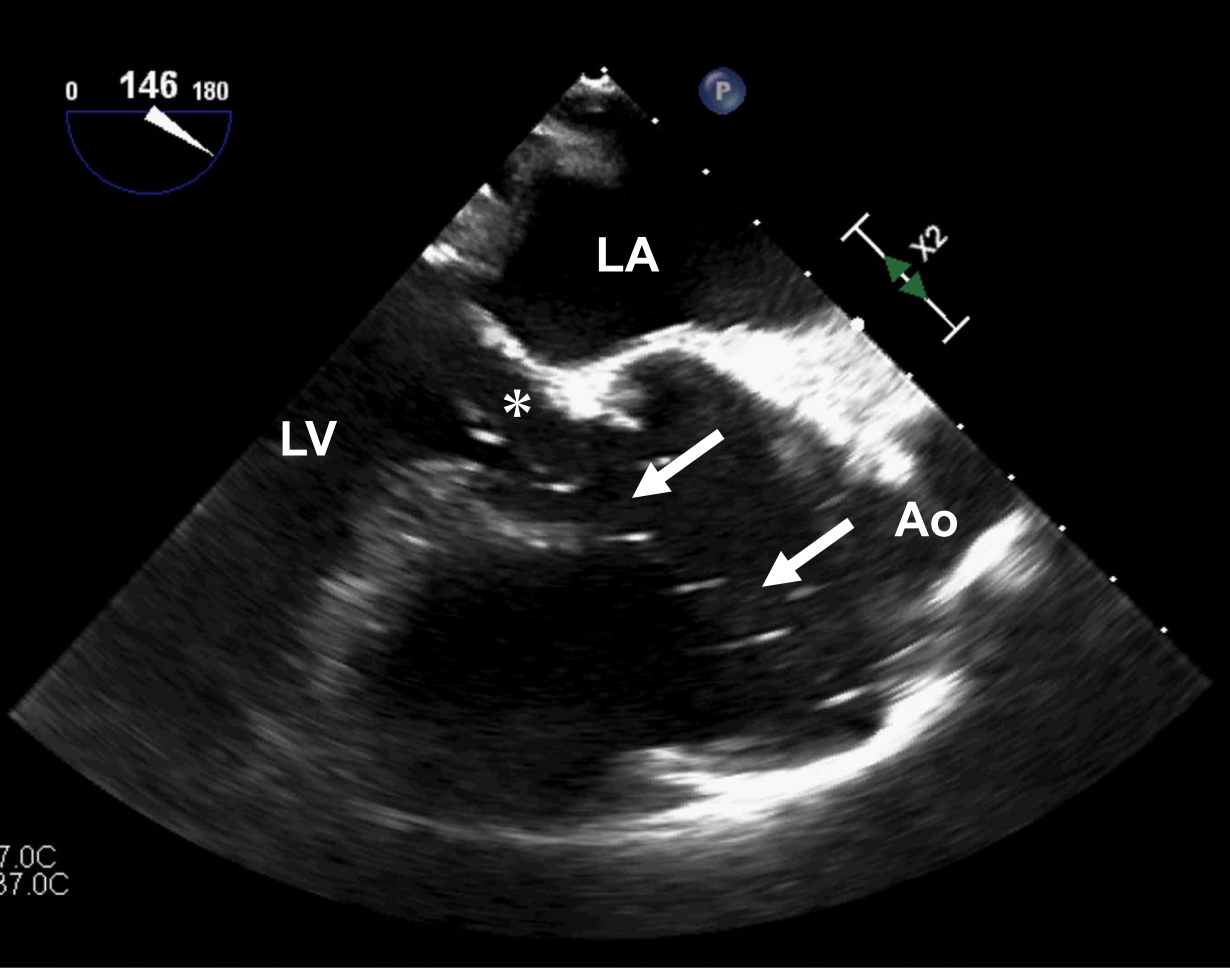
Echocardiographic mid-esophageal aortic valve long-axis view. The tip of the left ventricular vent (white arrow) was detected in the aortic root across the aortic valve (asterisk). Ao, ascending aorta; LA, left atrium; LV, left ventricle

**Fig. 3:**
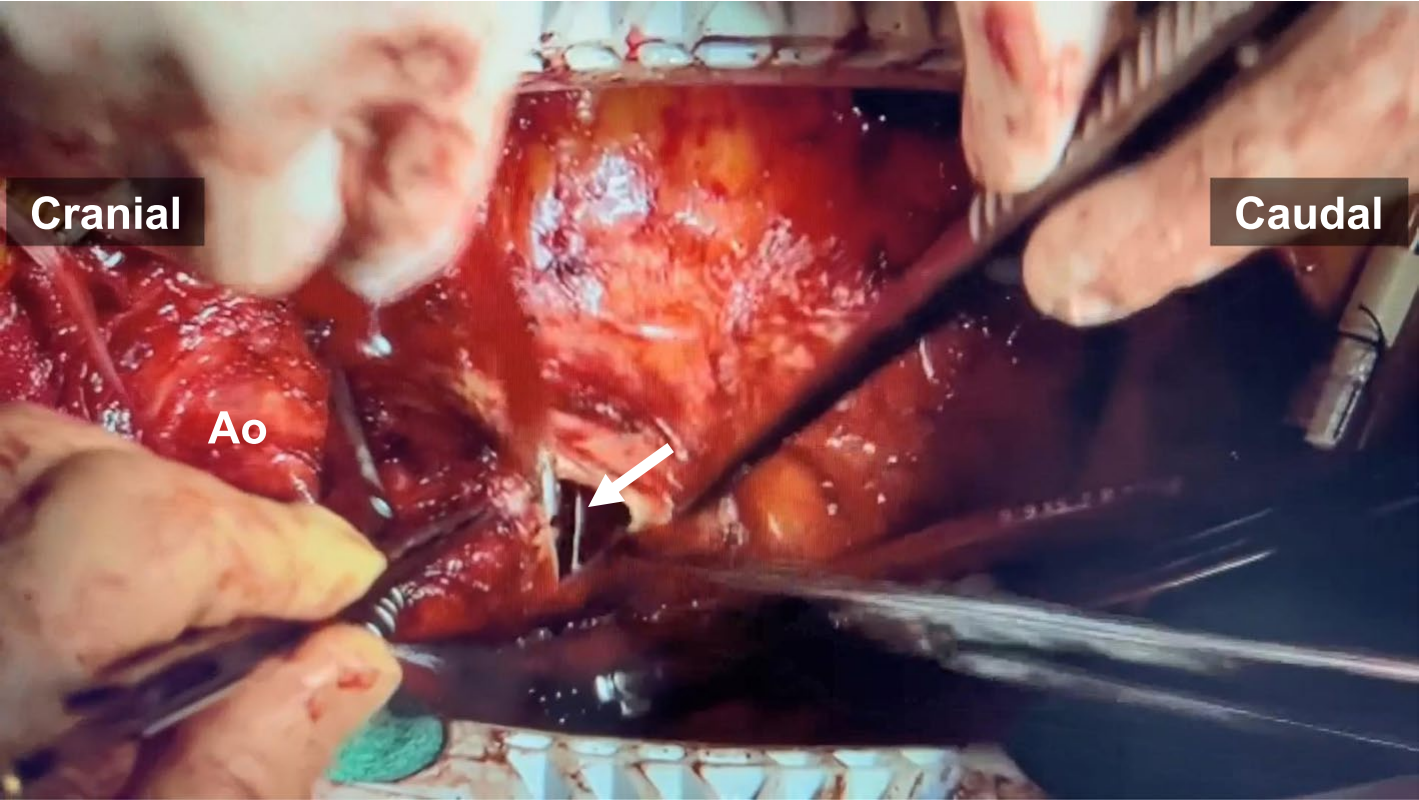
Image of the surgical field. The tip of the left ventricular vent was in the aortic root (white arrow), which was withdrawn to the left ventricular cavity under direct vision. Ao, ascending aorta

After starting cardioplegia, a large amount of clear fluid was drained from the LV vent; however, we could not detect AR by TEE. The cardioplegia flow and LV vent suction rates were increased. Despite delivery of two-thirds of the planned cardioplegia dose, an electrocardiogram did not result in complete cardiac arrest. Furthermore, TEE examination revealed that the tip of the LV vent had been misplaced into the aortic root across the aortic valve (Fig. 2). An electrocardiogram showed ventricular fibrillation; thus, the ascending aorta was incised, and additional cardioplegia was selectively delivered to the coronary ostia, resulting in cardiac arrest. During this process, the LV vent at the aortic root was withdrawn under direct vision to the LV cavity (Fig. 3). Then, 750 mL of blood cardioplegia (1:1 mixture of blood and crystalloid cardioplegic solution) was infused every 30 min at a rate of 200 mL/min in a selective antegrade fashion.

The Bentall procedure was performed without complication, and the patient was weaned off CPB without difficulty. The duration of the operation, CPB, and aortic cross-clamping time was 402, 224, and 156 min, respectively. After surgery, the patient was managed in the intensive care unit with tracheal intubation. The trachea was extubated on postoperative day 4. No postoperative complications were noted.

## Discussion

The LV vent is commonly inserted via the RSPV during cardiac surgery; however, LV vent insertion is associated with severe complications, such as systemic air embolism [1], bleeding, and injury to the heart, including late LV aneurysms [3]. Other risks of LV vent insertion include misplacement into the right inferior pulmonary vein, left pulmonary vein, or the left atrial appendage, which make the LV vent less effective [2]. In our case, the LV vent was inserted via the RSPV and then passed through the mitral and aortic valves into the aortic root.

Antegrade cardioplegia failure caused by misplacement of the LV vent is an unusual complication and has rarely been reported. Talwar et al. [4] reported a rare case of antegrade cardioplegia failure caused by accidental passage of the LV vent across the aortic valve, thus producing acute AR. Talwar et al. [4] noticed a rigid cord-like structure was palpated in the ascending aorta and speculated that the LV vent was inserted into the aortic root. In contrast, we diagnosed misplacement of the LV vent in the aortic root by TEE.

In our case, AR was initially thought to be the main cause of cardioplegia failure; however, we could not detect AR by TEE. The cardioplegia injected through the aortic root cannula likely drained through the LV vent that had been misplaced into the aortic root. AR and inadequate aortic cross-clamping are well-known reasons for cardioplegia failure [5]; however, it may be prudent to include LV vent misplacement into the aortic root.

There were three possible reasons why the LV vent passed through the aortic valve. First, geometric changes in the heart caused by enlarged aortic root may be the cause of misplacement. Enlargement of the heart chambers is known to pose a technical challenge for LV vent insertion [2]; therefore, care must be taken to compensate for geometric changes of the heart when inserting an LV vent. Second, poor surgical palpation due to adhesions caused by revision cardiac surgery may be the cause of misplacement. Indeed, it is difficult to insert the LV vent during cardiac revision and minimally invasive cardiac surgery because the surgeon’s hand cannot be placed behind the heart [2]. Finally, the insertion depth may be the cause of misplacement. In cases in which the LV vent is inserted too deeply, the LV vent might be pinched together during aortic cross-clamping. Deep insertion can also cause an LV laceration due to endocardial compression, so awareness of the LV vent depth is essential [6].

Placement of the LV vent in the LV cavity is usually confirmed by observing ventricular arrhythmias and regurgitation from the LV vent; however, these findings do not guarantee successful placement. The position of the tip must be confirmed by TEE to prevent severe complications [3]. The mid-esophageal four-chamber view is often used to confirm LV vent position beyond the mitral valve [7]; however, it is difficult to visualize the tip because the direction of the ultrasound beam and the direction of LV vent insertion are nearly the same. The strongest echoes are produced when the ultrasound beam angles of incidence to the LV vent approach the angle of reflection [8]. In trans-gastric two-chamber or trans-gastric apical short-axis views, the direction of the ultrasound beam and the direction of LV vent insertion are nearly perpendicular; therefore, it may be easier to visualize the tip. Accurate confirmation of the position of the LV vent tip with appropriate TEE views is important.

In conclusion, it is important to confirm the position of the tip by TEE to prevent severe complications associated with the LV vent.

## Supplementary Information

**Additional file 1: Video 1.** Transesophageal echocardiography finding.

**Additional file 2: Video 2.** Intraoperative finding.

## Data Availability

The datasets are available from the corresponding author on reasonable request.

## Abbreviations

LV: Left ventricular
RSPV: Right superior pulmonary vein
TEE: Transesophageal echocardiography
AR: Aortic regurgitation
CPB: Cardiopulmonary bypass
